# Large Scale Genotype Comparison of Human Papillomavirus E2-Host Interaction Networks Provides New Insights for E2 Molecular Functions

**DOI:** 10.1371/journal.ppat.1002761

**Published:** 2012-06-28

**Authors:** Mandy Muller, Yves Jacob, Louis Jones, Amélie Weiss, Laurent Brino, Thibault Chantier, Vincent Lotteau, Michel Favre, Caroline Demeret

**Affiliations:** 1 Unité de Génétique, Papillomavirus et Cancer Humain (GPCH), Institut Pasteur, Paris, France; 2 University Paris Diderot, Sorbonne Paris cite, Cellule Pasteur, Paris, France; 3 Groupe Logiciels et banques de données, Institut Pasteur, Paris, France; 4 HTS platform, CEBGS-IGBMC, Illkirch, France; 5 IMAP team, IFR-128 Biosciences, Lyon, France; Harvard Medical School, United States of America

## Abstract

Human Papillomaviruses (HPV) cause widespread infections in humans, resulting in latent infections or diseases ranging from benign hyperplasia to cancers. HPV-induced pathologies result from complex interplays between viral proteins and the host proteome. Given the major public health concern due to HPV-associated cancers, most studies have focused on the early proteins expressed by HPV genotypes with high oncogenic potential (designated high-risk HPV or HR-HPV). To advance the global understanding of HPV pathogenesis, we mapped the virus/host interaction networks of the E2 regulatory protein from 12 genotypes representative of the range of HPV pathogenicity. Large-scale identification of E2-interaction partners was performed by yeast two-hybrid screenings of a HaCaT cDNA library. Based on a high-confidence scoring scheme, a subset of these partners was then validated for pair-wise interaction in mammalian cells with the whole range of the 12 E2 proteins, allowing a comparative interaction analysis. Hierarchical clustering of E2-host interaction profiles mostly recapitulated HPV phylogeny and provides clues to the involvement of E2 in HPV infection. A set of cellular proteins could thus be identified discriminating, among the mucosal HPV, E2 proteins of HR-HPV 16 or 18 from the non-oncogenic genital HPV. The study of the interaction networks revealed a preferential hijacking of highly connected cellular proteins and the targeting of several functional families. These include transcription regulation, regulation of apoptosis, RNA processing, ubiquitination and intracellular trafficking. The present work provides an overview of E2 biological functions across multiple HPV genotypes.

## Author Summary

Over 100 types of human papillomaviruses are responsible for widespread infections in humans. They cause a wide range of pathologies, ranging from inapparent infections to benign lesions, hyperplasia or cancers. Such heterogeneity results from variable interplay among viral and host cell proteins. Aiming to identify specific features that distinguish different pathological genotypes, we mapped the virus-host interaction networks of the regulatory E2 proteins from a set of 12 genotypes representative of HPV diversity. The E2-host interaction profiles recapitulate HPV phylogeny, thus providing a valuable framework for understanding the role of E2 in HPV infection of different pathological traits. The E2 proteins tend to bind to highly connected cellular proteins, indicating a profound effect on the host cell. These interactions predominantly impact on a subset of cellular processes, like transcriptional regulation, apoptosis, RNA metabolism, ubiquitination or intracellular transport. This work improves the global understanding of HPV-associated pathologies, and provides a framework to select interactions that can be used as targets for the development of new therapeutics.

## Introduction

Papillomaviruses are non-enveloped small DNA viruses, of which over 140 types infect humans (HPV). HPV are strictly epitheliotropic, with specificity for stratified epithelia of the skin (cutaneous HPV) or genital and oral mucosa (mucosal HPV). They are either associated with asymptomatic infections or induce benign proliferative lesions, which have the potential to progress toward malignancy for the ‘high risk’ HPV (HR-HPV). Although carcinogenic conversion occurs only in a minority of infections, mucosal HR-HPV are associated with almost all cervical cancers, and with 50% anogenital and 30% head and neck cancers [1]. In addition, growing evidence point to a role of some cutaneous HPV in non-melanoma skin cancer [2]. Therefore, from inapparent infections to cancers, HPV cover a large spectrum of diseases in humans [3].

The productive viral cycle both depends on and perturbs the differentiation of infected keratinocytes [4], and HPV pathogenesis relies on complex interplay between early viral and host proteins. The carcinogenic conversion of HR-HPV-associated lesions proceeds from a deregulation of virus-host cross-talk, leading to over-expression of E6 and E7 viral oncogenes and to the accumulation of cellular genetic alterations. This long-lasting process culminates in the emergence of fully-transformed cells critically dependent on the immortalizing properties of the HR-HPV E6 and E7 proteins to drive continuous cell proliferation.

The HPV E2 early protein is a pivotal factor of both productive and persistent infection. It provides the control of viral DNA transcription, replication and mitotic segregation through specific binding to the viral genome. Such activities are shared by all HPV and are mediated by E2 interactions with cellular transcription factors, mitosis-associated factors, and with the viral E1 helicase (see [5], [6] for review). As such, the E2 protein is mainly envisioned as a basic viral factor. Contrary to the E6 and E7 proteins, the involvement of E2 in the different features of HPV pathology is elusive. Indeed, only few studies demonstrated that E2 functions may differ between oncogenic HR-HPV and the Low-Risk HPV (LR-HPV), which are always associated with benign hyperplasia. Some activities are specific of the HR-HPV E2 proteins, such as the induction of apoptosis or of a G2/M cell cycle arrest [7]–[9]. In addition, the HR-HPVE2 proteins induce genomic instability [9], and E2 from cutaneous HPV8 exhibits intrinsic oncogenic potential when expressed in the skin of transgenic mice [10], pointing to a role of E2 in the carcinogenic conversion of HR-HPV associated lesions (see [11] for review).

Given the major public health concern caused by genital cancers, the activities of viral early proteins have been far more extensively studied for mucosal HR-HPV than for other HPV. However, the variability of HPV-associated lesions indicates that the interplay among viral and host proteins may strongly differ. A global understanding of cell alterations generated by viral proteins according to the tropism and pathogenic potential is currently lacking. To make progress in this issue, we mapped the virus-host protein-protein interactions of the E2 proteins from 12 genotypes representative of HPV diversity. We selected HPV of different tropism specificity (cutaneous: HPV1, 3, 5, 8, 9 or mucosal: HPV6, 11, 16, 18, 32, 33, 39) and with different pathogenic potential (LR-HPV 1, 3, 6, 9, 11, 32 or HR-HPV 5, 8, 16, 18, 33, 39). This selection spans over three clades of the typical HPV phylogeny based on the sequence of the L1 capsid protein [12]: α-types HPV 3, 6, 11, 16, 18, 32, 33, 39; β-types HPV 5, 8, 9 and μ-type HPV 1. Interaction mapping was performed by combining a large scale identification of E2 partners by successive yeast two-hybrid screenings and a cell-based interaction assay for the validation of protein-protein interactions. This work gives an overview of E2 biological functions across multiple HPV genotypes, and provides a comprehensive framework for understanding the role of E2 in HPV pathologies.

## Results/Discussion

### Mapping of E2-Host Protein-Protein Interactions by Yeast Two Hybrid Screenings

To provide a comprehensive assessment of E2-host Protein-Protein Interactions (PPI), we mapped PPI of E2 from 12 HPV genotypes representative of HPV tropism and pathogenic potential: mucosal HR-HPV 16, 18, 33 and 39; mucosal LR-HPV 6, 11 and 32; cutaneous HPV 1, 3, 5, 8 and 9. The 12 E2 proteins were used as baits in a mating-based yeast two-hybrid (Y2H) to screen a human keratinocyte (HaCaT) cDNA library. The number of diploid yeasts generated was systematically evaluated to be at least ten times higher than the library complexity. In order to obtain exhaustive Y2H datasets, successive screenings were performed with each of the E2 proteins.

In total, the Y2H screen identified 251 distinct interactions involving 202 different cellular proteins. Few proteins were interacting with numerous E2, indicating a low overlap of E2-PPI in Y2H. Indeed, only 27 proteins (13.4%) were picked up with more than one E2 protein as follows: 16 with two E2, seven with three E2, and a single one with four, five, six or seven E2 proteins. Five proteins (GPS2, SFRS1, AP3D1, C1QBP and TP53) had been previously identified as E2 interacting proteins in the literature (further referred to as Literature Curated E2-Protein Protein Interactions or LCE2-PPI). LCE2-PPI were extracted from the VirHostNet [13], virusMINT [14] and PubMed databases (Table S1). For some of these interactors, PPI were detected in our Y2H screen with an E2 protein of a different HPV genotype than in LCE2-PPI (Table S2). These latter cases probably point to shared E2-PPI, which could be verified through their assessment with the series of 12 E2 proteins. The recovery of 5 out of 53 known E2 partners indicated a sensitivity of Y2H screening around 10%, which is in the range of previously described similar analyses [15]. This, combined with the high coverage of the HaCaT cDNA library reached in each screening, suggest a satisfactory sampling sensitivity. Overall, the Y2H screen led to the identification of 197 new potential cellular binding partners of at least one E2 protein.

### Matrix Building for the Validation of E2-Host PPI

The Y2H screen applied to a wide spectrum of HPV genotypes was appropriate to get an overview of E2-PPI without bias toward the most studied E2 proteins, contrary to the LCE2-PPI datasets. However, the coverage of PPI detected by Y2H is estimated to be around 20% of total PPI [16], highlighting a high false-negative rate inherent to this screening methodology [17]. We therefore speculated that, despite repetitive probing of the HaCaT cDNA library with each of the E2 protein, PPI detected with a subset of E2 might have escaped detection with the others, which would explain the low overlap of E2-PPI observed in the Y2H screens. Moreover, it was previously demonstrated that combining different methodologies is necessary to increase the robustness of PPI datasets [18]. A stringent validation strategy consists in the use of orthogonal PPI detection methods, as it ensures the discarding of false positive interactions generated in Y2H screens.

**Figure 1:**
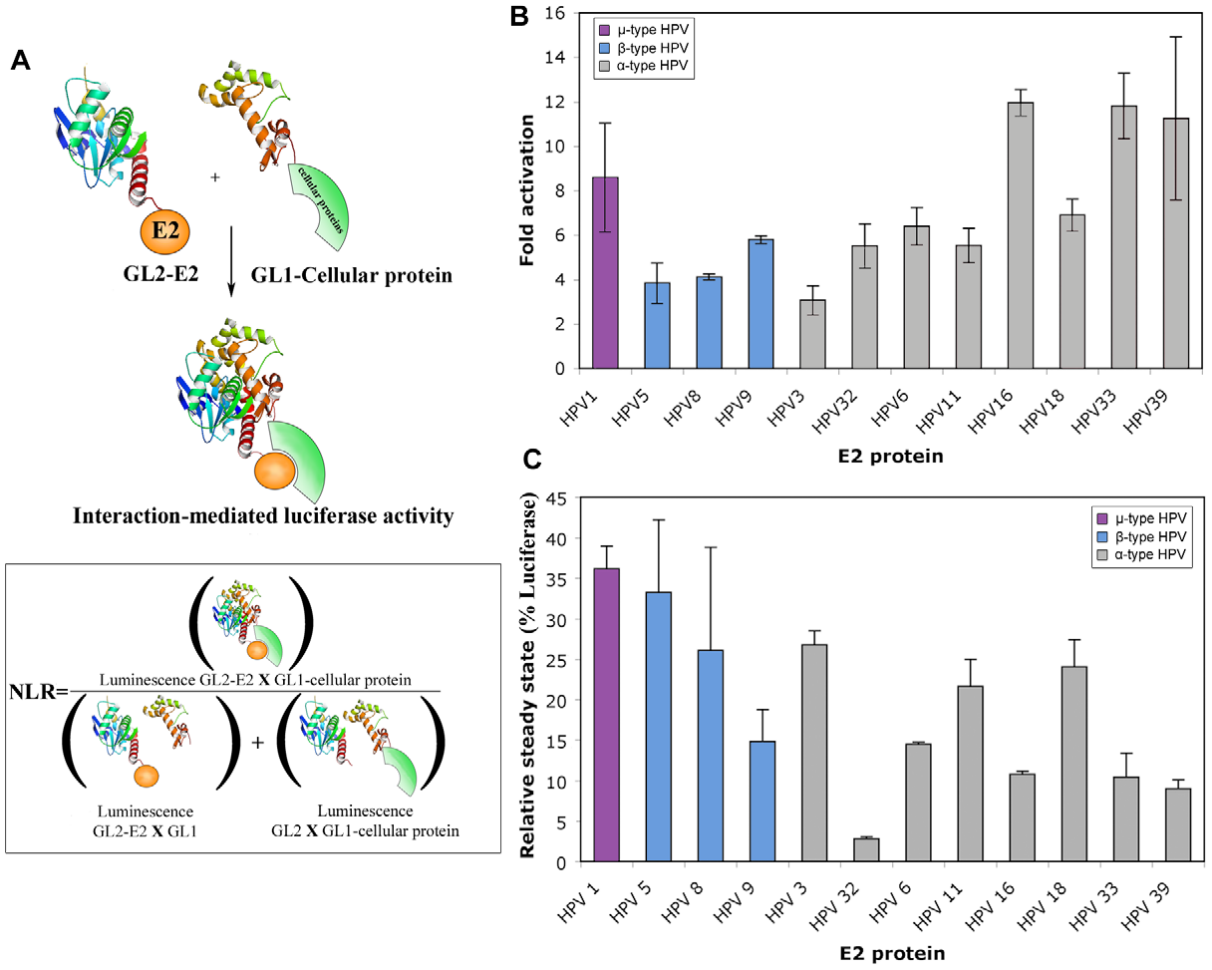
Characterization of E2 proteins expressed in HT-GPCA conditions. (A) Schematic representation of the HT-GPCA method. This assay is based on the reconstitution of a luciferase activity upon co-expression of interacting partners in fusion with two inactive fragments of the *Gaussia princeps* luciferase (designated GL1 and GL2). The reconstituted Luciferase activity is estimated from a Normalized Luminescence Ratio (NLR) (B) 293T cells were transfected with the pTK6E2BS-Luc reporter and the GL2-E2 expressing plasmids. Fold activation is given relative to TK6E2BS-Luc in absence of E2. (C) E2-Firefly luciferase fusion proteins were expressed in 293T cells and the firefly luciferase activity was determined 24 h post-transfection. The results are expressed as a percentage of the activity obtained with the firefly luciferase only.

We thus decided to challenge a subset of cellular proteins selected from the Y2H screen for pair-wise interaction with the whole set of the 12 E2 proteins using a mammalian cell-based orthogonal PPI detection assay. Such a strategy allows a comparative interaction analysis among the different HPV genotypes. We used a secondary High-Throughput *Gaussia princeps* luciferase-based Complementation Assay (HT-GPCA, Figure 1A) recently described [19]. Briefly, bait and prey proteins were expressed in 293T cells in fusion with two inactive fragments of the *Gaussia princeps* luciferase (designated GL1 and GL2), which restore a significant enzymatic activity when brought in close proximity by an interaction. The reconstituted Luciferase activity is estimated from a Normalized Luminescence Ratio (NLR, Figure 1A). This assay has been recently benchmarked by using two positive reference sets of protein pairs known to interact, and a set of *a priori* non-interacting protein pairs [19]. It was determined that when setting a NLR threshold of 3.5, there was only 30% false negatives (known PPI not recovered in HT-GPCA) and 2.5% false positives. A 3.5 NLR threshold was accordingly used to discriminate positive interactions in the present study.

A high-confidence core set of 48 potential E2 partners was selected for validation from the Y2H dataset by keeping proteins identified at least three times in Y2H [20]. Assuming that potential false positives would be eliminated by combining two orthogonal methods, 54 proteins found only one or two times were additionally rescued for further validation in mammalian cells. This non-core set consisted in proteins known from other studies as E2 partners, proteins functionally relevant to E2 (transcription, replication factors), or proteins related to potential E2 partners of the core set. In total, 102 proteins were selected, corresponding to 138 distinct Y2H interactions obtained through 1,135 sequenced PPI (Figure S1 and Table S3). We also increased the explored area by including 19 known E2 partners, which were used as positive controls herein referred as Gold Standards (GS). Combined with the five known E2 partners recovered in our Y2H screen, the final list of GS comprised 24 cellular proteins. In total, 121 cellular proteins were to be validated for interaction with E2, of which 97 represented novel potential partners of E2.

Before proceeding to HT-GPCA, we wished to ensure that fusion with a Gaussia fragment would not alter the folding and functionality of E2 in the GL2-E2 fusion proteins. To that aim, we assessed E2-dependent transcription of pTK6E2BS, containing six E2 binding sites (E2BS) upstream of the minimal TK promoter. The sequences of E2BS were designed to be optimal for the binding of a large panel of E2 [21] in order to homogenize E2 binding to this promoter. All GL2-E2 fusion proteins properly activated transcription, demonstrating that the E2 proteins were functional (Figure 1B) and thus that fusion of the GL2 tag at their N-terminus did not induce incorrect folding or localization. The relative accumulation of the E2 proteins was approximated by fusion with the *Firefly* luciferase protein (Fluc-E2 fusion), so that their expression levels could be deduced from luciferase activity as previously reported [22]. Fluc-E2 fusion proteins accumulated to levels ranging from 5% (HPV32 E2) to 35% (HPV1 E2) of the Firefly luciferase alone, indicating variations in E2 accumulation levels (Figure 1C). However, there was no correlation between steady-state levels and transcriptional activation (Figure 1B and 1C), pointing to differences in the intrinsic transcriptional properties of the E2 proteins, thereby corroborating previous studies [23], [24]. As for the GL2-E2 fusion proteins, the expression levels of the selected 121 cellular proteins expressed as GL1 fusions may vary. The heterogeneity in protein accumulation levels would potentially bring a degree of variability in HT-GPCA assay, that have to be taken into consideration for the comparative analysis of their interaction patterns.

### Analysis of the Interactions between E2 Proteins and the Gold Standards by HT-GPCA

**Figure 2:**
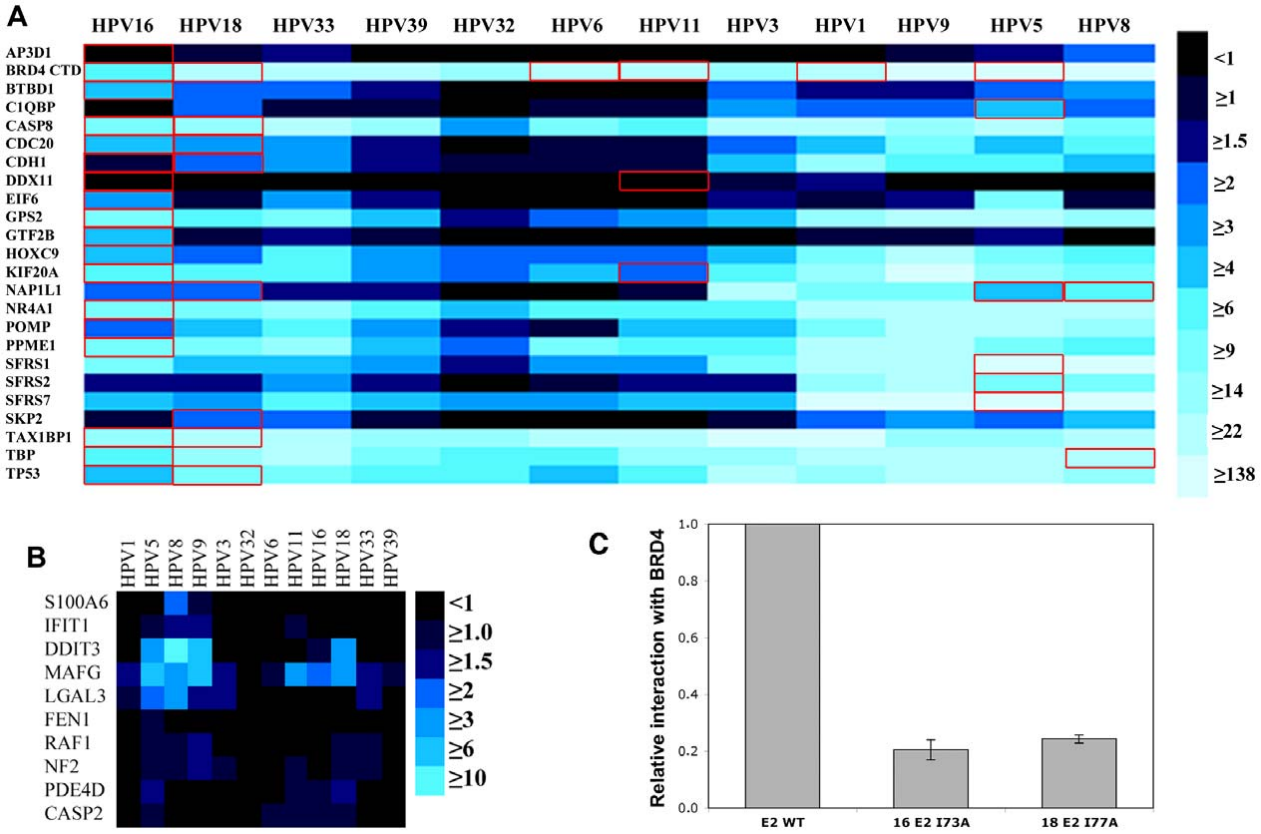
Interaction of E2 with gold standards by HT-GPCA. (A) Heat maps representing the interactions between the 12 E2 proteins (by columns) and the gold standards (rows). The colour represents Normalized Luminescence Ratio (NLR) obtained by HT-GPCA, from no interaction (black) to strong interactions (light blue). The red rectangles indicate interactions identified in the literature (LCE2-PPI) (B) Heat maps representing the interactions between the 12 E2 proteins (by columns) and the negative random set (rows). (C) Interaction between BRD4 CTD and mutated E2 proteins (16E2I73A and 18E2I77A) tested by HT-GPCA. The results are displayed relative to BRD4 CTD interaction with the wild-type E2 proteins.

To evaluate the reliability and sensitivity of the HT-GPCA method applied to E2, we first conducted a pilot experiment with the set of 24 gold standards (GS), which covered about half of LCE2-PPI (Table S1). The results are displayed as Heat maps where the intensity of an interaction, based on the NLR, is represented by a color gradient from black (no interaction) to light blue (strong interaction) (Figure 2A). As underscored in Figure 2, the majority of E2-PPI in the GS set had been studied with a single E2, mainly 16E2. Thus our approach significantly broadens the scope of GS analysis.

Out of the 39 studied known E2 interactions (LCE2-PPI), 28 interactions (72%) were recovered by HT-GPCA (Figure 2A and Table S4). For 7 of the 11 expected LCE2-PPI that failed to be recovered, the corresponding gold standard protein interacted with other E2 proteins, suggesting that the missing interactions represent HT-GPCA false negative interactions. We noticed that PPI with p53 (TP53) were detected in HT-GPCA with all the E2 proteins, in contrast to previous studies showing that this interaction was restricted to mucosal HR-HPV E2 [8]. Such discrepancies might be due to increased sensitivity of HT-GPCA, and outline the need to combine different methods to improve the confidence of interactions datasets.

A negative control interaction matrix provided a rough estimate of the false-positive rate of PPI detected by HT-GPCA applied to the E2 proteins. It consisted in cellular proteins randomly picked in the human ORFeome resource, *a priori* not interacting with E2. Among this matrix of 120 PPI (12E2×10 proteins) the false positive rate was 5.8% (Figure 2B).

Furthermore, the specificity of HT-GPCA was illustrated by using E2 proteins invalidated for interaction with BRD4 by mutation of Isoleucine 73 (HPV16) or 77 (HPV18) to Alanine [25], [26]. Both mutated proteins, 16E2 I73A and 18E2 I77A exhibited an impaired binding to BRD4, with a five fold decrease of NLR when compared to the wild-type proteins (Figure 2C).

Overall, these data demonstrate both the robustness and the sensitivity of HT-GPCA to detect pair wise interactions involving the E2 proteins.

### Mapping of the 12 E2-Host Interaction Profiles by HT-GPCA

We then processed all selected cellular proteins, performing 1,452 ( = 121×12) tests. In total, 617 interactions (42%) exhibited a NLR above 3.5, thereby scoring positive in HT-GPCA. Of the 121 cellular proteins tested, 23 (19%) did not engage detectable interaction with any of the E2 proteins (Table S5). Of note, virtually all of the 98 validated partners interacted with more than one E2 protein, highlighting a high overlap between E2 interactors.

Comparison of the Y2H and HT-GPCA PPI datasets (schematized in Figure S1) indicated that among the 138 interactions detected in the Y2H screen (Y2H-PPI), 72 were validated in HT-GPCA, representing 53 cellular proteins. 38 Y2H-PPI, involving 27 cellular proteins, were not recovered in HT-GPCA but interactions were detected with different E2 proteins than in Y2H. As discussed previously, we assume that the corresponding non-recovered Y2H-PPI most probably represent HT-GPCA false negative interactions. Lastly, 28 Y2H-PPI were not validated and involved 22 cellular proteins that did not interact with any E2 proteins. These proteins were consequently discarded for further analyses. Altogether, these results point a 53% overlap between Y2H-PPI and HT-GPCA interactions. When considering the interactors, the recovery was 79%.

**Figure 3:**
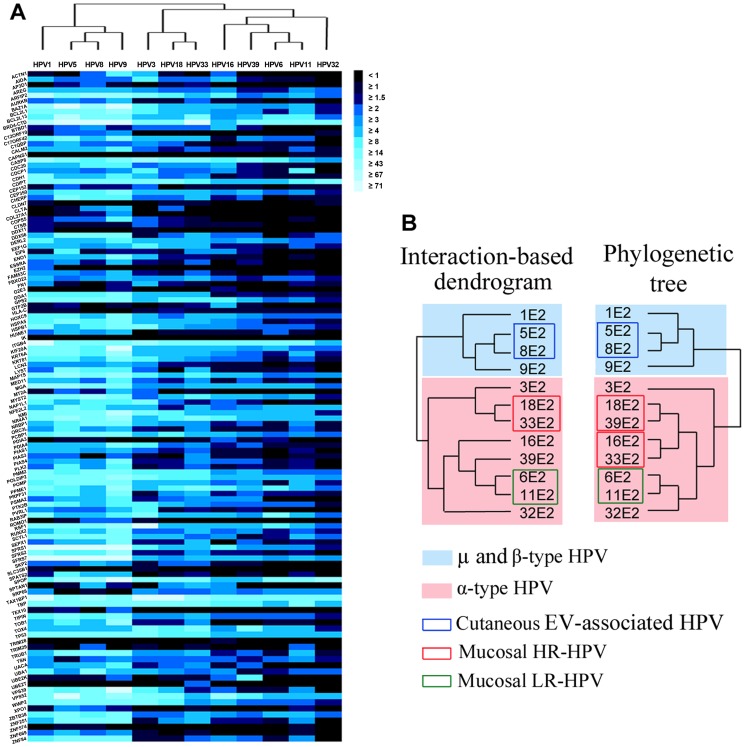
Interaction map between the 12 E2 proteins and the 121 cellular proteins by HT-GPCA and hierarchical clustering. (A) Heat maps representing the complete dataset of interactions between the 12 E2 proteins (by columns) and the 121 cellular proteins (by rows). The intensity of interaction is represented by the colour, from black (no interaction) to light blue (strong interactions) based on Normalized Luminescence Ratio (NLR). The E2-PPI profiles were clustered according to their similarities by hierarchical clustering (tree above the heat map). (B) Interaction dendrogram generated from the hierarchical clustering of E2-interaction profiles and phylogenetic tree based on E2 sequences alignment.

PPI validation rate was higher with μ- or β-types E2 proteins (HPV1, 5, 8, 9) than with the α-types E2 (HPV 3, 6, 11, 16, 18, 32, 33 and 39), as reflected by brightness variations of the heat maps (Figure 3A). Significantly, the overall NLR levels were not related to E2 accumulation levels, since 9E2 exhibited the highest interaction rate but was not the most accumulated. Conversely, 33E2 engaged the most interactions in the mucosal group, whereas it accumulated at low levels (Figure 1C). These observations clearly argue that variations in E2 accumulation levels are not driving the differences observed by HT-GPCA, and therefore do not essentially alter this comparative interaction mapping. Differences in E2 interaction rates are more likely related to intrinsic characteristics of the proteins. Notably, the μ or β-E2 proteins contain the longest hinge regions (> 122 amino acid, <79 for the others), which is an intrinsically disordered segment in E2 proteins. Their higher interaction rate is consistent with the notion that disordered regions are enriched in exposed interaction motifs [27].

The E2-host PPI profiles were gathered according to their similarities by agglomerative hierarchical clustering (Cluster software). Matrix tree plots were generated from this analysis, and were used to build an E2-interaction dendrogram using the UPGMA (Unweighted Pair Group Method with Arithmetic Mean) method with the Euclidian distance function and a complete linkage method (Figure 3B). Strikingly, in the interaction dendrogram, the E2 proteins segregated according to HPV tropism (cutaneous and mucosal), and further clustered following pathogenic potential (high-risk versus low-risk). We found a high correlation between the E2-interaction dendrogram and the phylogenetic dendrogram based on the E2 sequences (Figure 3B), the Pearson correlation coefficient calculated from the distance matrices of the two dendrograms was 0.91, with a p-value < 10^−10^. Of note, using different parameters for the clustering analysis did not drastically affect the structure of the interaction dendrogram or the closeness to phylogenetic tree (Figure S2). This observation demonstrates the robustness of the interaction dendrogram generated by our approach.

The E2 protein is mainly envisioned as a basic viral factor, essential for all HPV through its regulatory role of viral DNA transcription, replication and mitotic segregation. Only few studies demonstrated that E2 activities may differ according to HR or LR-HPV type [8], [9], [27].

We show in the present study that the E2 proteins engage different patterns of interaction with the host proteome depending on both the tropism and the HR or LR trait of HPV. Such interaction mapping may thus improve the understanding of cell alterations induced by E2.

### E2-PPI in Correlation with the HR Trait of HPV 16 and 18

The very first branching division in the E2-PPI dendrogram separates the β/μ from the α-types E2, which essentially corresponds to a distinction between cutaneous (β/μ-HPV) and mucosal (α-HPV except for HPV3) HPV. Within each group, the interaction profiles further clustered according to pathogenic potential.

Now considering only the α group, we compared the interaction profiles of E2 from the genital HR-HPV 16, 18, 33 and 39 with those of the LR-HPV 6 and 11, in order to extract interactions that may play a role for the life cycle of mucosal HR-HPV. Only one protein, GPS2, which is an integral component of the NCoR complex (Nuclear receptor Co-Repressor) [28] interacted with all mucosal HR-HPV and not with the LR-HPV E2 proteins. Focusing on the most prevalent HR-HPV16 and 18, we identified a series of cellular proteins differentially bound by either 16E2 or 18E2 compared to the LR-HPV E2 proteins (table 1). Six of these proteins were targeted by both 16E2 and 18E2 (GPS2, HSP5A, ARFIP2, CDC20, SPTAN, VPS52), whereas the others were genotype-specific. It is noteworthy that the above-mentioned cellular partners interact with members of the μ/β-types HPV E2 proteins, suggesting that they could also take part to HPV pathogenesis in the context of cutaneous tropism. Of note, the CDC20 protein was recovered among the HR-specific partners interacting with both 16 and 18E2, in line with the proposed role of this interaction in carcinogenic conversion associated with both genotypes [9]. Given that the mucosal HR and LR viruses infect distinct biological niches (HPV16/18 infect mucosal transformation zones, while HPV6/11 infect the external genitalia), such discriminating interactions could result from the different proteome of infected tissues. They nevertheless might point to targets important for the life cycle of HPV16 or HPV18 genotypes, responsible for most of the genital cancers.

**Table 1:** HR-specific interactions of the E2 proteins from HPV16 and HPV18.

	Protein name
**HPV 16 and 18 E2**	ARFIP2, CDC20, GPS2, HSPA5,SPTAN1,VPS52
**HPV16 E2**	AIDA, ARFIP2, BTBD1, CDC20, EIF6, GPS2, GTF2B, HOXC9, HSPA5, MGA, PDIA3, PSMA2, SFRS1, SPOP *, SPTAN1, TOX4*, VPS52
**HPV 18 E2**	ARFIP2, CASP8, CDC20, CLTA,DERL2,EEF1G, GGA1,GPS2,HSPA5, HSPB1, KRT6A, KRT81,MAP1S, MYST2, NMI, PCBP1, PMM2, PRPF31, PTK2B, SCYL1, SKP2, SPTAN1, TOX4, VPS39, VPS52, WWP2

List of the cellular proteins involved in interactions discriminating the E2 proteins of the genital HR-HPV 16 and 18 from the LR-HPV 6 and 11, based on the NLR profiles obtained by HT-GPCA. The asterisks (*) stand for cellular proteins generating lower NLR specifically with 16 E2 protein.

### Topological Analysis of the E2-Host Interaction Networks

Topological analysis of viral interaction networks can be informative with regard to the global impact of viral proteins in the host cell, as well as the dynamics of viral pathogenesis. To conduct such analysis, we built E2-host interaction networks with PPI scoring positive in HT-GPCA.

**Figure 4:**
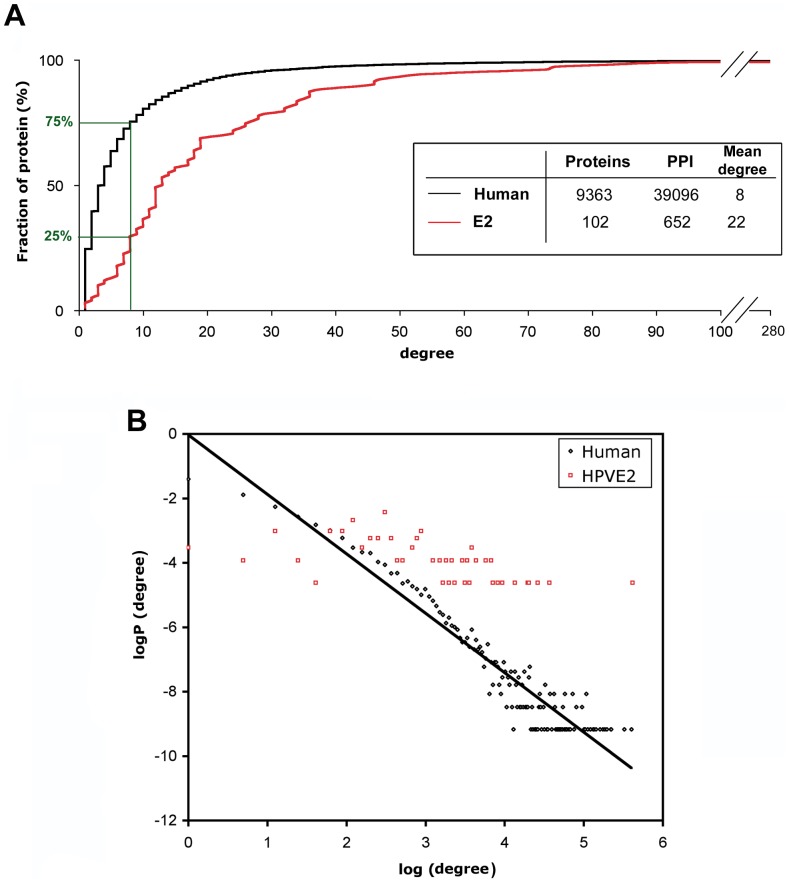
Topological analysis of the E2 interaction network. (A) Cumulative distribution of node degree of a reconstructed human interactome (black curve) and the E2 interactome (red curve). The fraction of proteins under the estimated average degree of the human interactome (8) is represented. The characteristics of each interactome are given in the inset. (B) Distribution of degree probability of the human (black) and the E2 interactome (red). P(degree) is the probability to connect K other proteins in the network. For the human interactome, the straight line represents the linear regression fit of the data (with a correlation coefficient R^2^ = 0.91). For the E2 interactome, we could not fit the data to a linear regression (R^2^ = 0.34).

The degree of a protein reflects the number of interactions it engages in the cell, and the degree distribution of a network gives a measure of its local dynamics. We studied the degree distribution of the E2-host network compared to that of a human interactome reconstructed from Human Protein Reference Database (HPRD 2010 release 9), including 39,100 binary protein-protein interactions. The cumulative plot of E2 and human interactomes relative to protein degree (Figure 4A) shows that 75% of proteins of the human interactome have a degree lower than eight (estimated mean degree of the present human interactome), while for only 25% of E2 targets the degree is lower than eight. Such difference was found statistically significant by the Kolmogorov-Smirnov test (0.5 with a p-value < 0.002). These results indicated that E2 proteins preferentially bind to highly connected proteins, also called hubs. The distribution of degree probability in both interactomes further substantiates a clear overrepresentation of high-degree proteins in the E2 interactome (Figure 4B). Overall, these results show that E2 proteins preferentially target highly connected cellular proteins. Such findings indicate that E2 broadly impacts on host cells by interacting with key proteins involved in many pathways of the cellular network. This likely maximizes E2 effects on a wide range of cellular functions. The preferential targeting of central proteins was previously observed with other viral proteins from EBV, KSHV and HCV [29]–[31]. Indeed, the binding to hub proteins could be a general hallmark of viral proteins to hijack at a systemic level the cellular interactome. It is noteworthy that protein centrality has been correlated with the presence of disordered regions [27]. We may therefore speculate that the intrinsically disordered hinge region provides a platform for most E2 interactions with the host proteome, as discussed previously.

### Functional Enrichment Analysis of the E2-Host Interaction Networks

**Figure 5:**
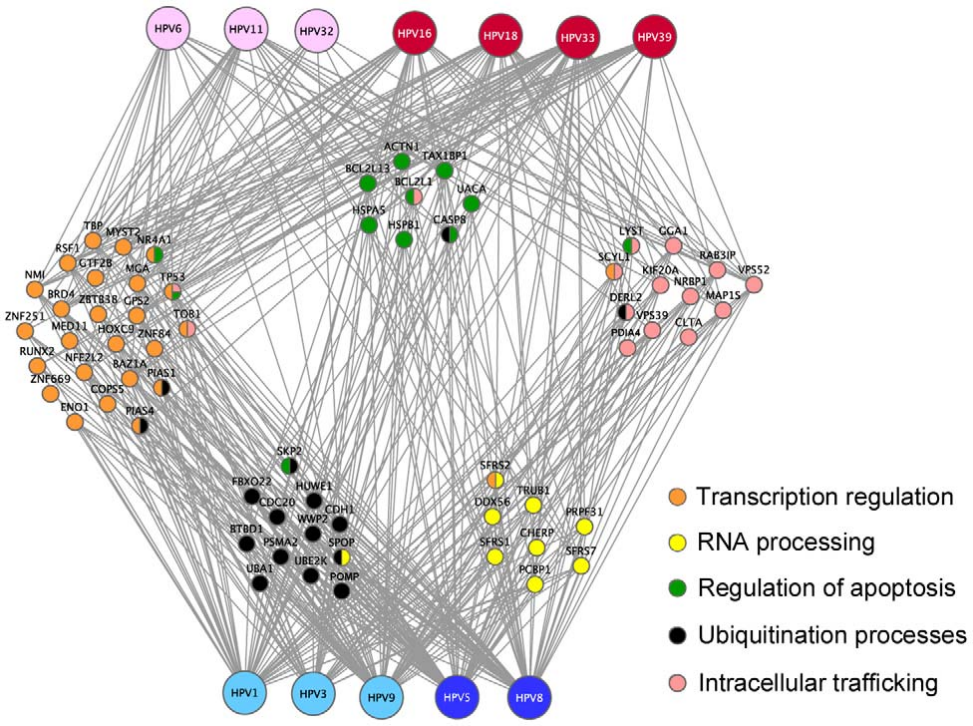
E2-targeted functional families. Cellular proteins (nodes) classified into enriched families based on the Gene Ontology annotations are colored according to the associated GO functions. Proteins shared by different families are bi-coloured. The network representation was generated by Cytoscape.

We next analyzed the E2 interaction network from a functional point of view to get insights into the functions of E2 that could emerge. Gathering of E2-targeted cellular proteins based on their GO (Gene Ontology) terms with the DAVID bioinformatics base [32] indicated enrichment of E2 targets in the following functional families: regulation of transcription, regulation of apoptosis, RNA processing, ubiquitination processes and intracellular transport (Figure 5 and details of the DAVID analysis are given in the Figure S3). This analysis gives an overview of E2 biological functions across multiple HPV genotypes.

#### Transcription regulation

The most significant targeted category consisted of proteins involved in transcriptional regulation, corroborating the prominent role of E2 as a transcription factor. It also highlights both the reliability of our approach and the pertinence of the interaction datasets. This cluster is composed of 26 proteins including transcription factors and cofactors, activators and repressors (table 2 and table S6). Only seven factors were bound by almost all E2 proteins (BRD4, TBP, MGA, TP53, RSF, NMI and MYST2), and could be instrumental for E2 transcriptional activity. Non-shared partners would rather underlie some specificity in intrinsic transcriptional properties of the E2 proteins. For example, our interaction data indicate that the general transcription factor TFIIB (GTF2B) is only targeted by 16E2. This could account for the improved recruitment of basal transcription components, which has been proposed for this viral protein [23]. Of note, the equal proportion of E2-targeted factors involved in activation and in repression of transcription likely underlies a dual role of E2 in transcriptional regulation. It highlights the importance of repression in the transcriptional regulatory functions of E2. In line with this notion, a recent study showed that the recruitment of multiple repressors by HPV18 E2 is required for full repression of early promoter transcription [33]. Interestingly, of the 34 cellular proteins that were found involved in this repression, two (BRD4, HSPB1) have been detected as direct E2 targets in the present study. Overall, the E2 interaction mapping provides an experimental assessment of the complex interplay between the E2 proteins and the host cell transcriptional machinery.

**Table 2:** Functional enrichment analysis of E2 targets.

Family name	GO term	GO name	Number of proteins	p-value	Protein name
**Transcription**	0006350	Transcription	22	0.026	ZNF84, RSF1, NMI, COPS5, TP53, MED11, NR4A1, TBP, ZNF669, GTF2B, ZNF251, ZBTB38, HOXC9, BAZ1A, SCYL1, PIAS4, MGA, PIAS1, NFE2L2, MYST2, RUNX2, ENO1, BRD4*
	0003700	Transcription factor activity	10	0.115	HOXC9, TP53, MGA, NR4A1, TBP, NFE2L2, MYST2, RUNX2, ZBTB38, ENO1
	0003712	Transcription cofactor activity	8	0.005	NMI, COPS5, PIAS4, PIAS1, GPS2, TOB1, SFRS2, ENO1
	0016563	Transcription activator activity	7	0.032	RSF1, HOXC9, COPS5, NR4A1, PIAS1, NFE2L2, RUNX2
	0016564	Transcription repressor activity	7	0.010	RSF1, PIAS4, PIAS1, GPS2, TOB1, SFRS2, ENO1
**RNA processing**	0006396	RNA processing	9	0.025	SFRS7, PRPF31, DDX56, CHERP, PCBP1, SFRS1, TRUB1, SFRS2, SPOP
	0008380	RNA splicing	6	0.113	SFRS7, PRPF31, PCBP1, SFRS1, SFRS2, SPOP
**Apoptosis**	0042981	Regulation of apoptosis	12	0.015	UACA, LYST, CASP8, SKP2, TP53, NR4A1, HSPB1, ACTN1, BCL2L1, HSPA5, BCL2L13, TAX1BP1
	0043065	Positive regulation of apoptosis	8	0.022	UACA, LYST, CASP8, SKP2, TP53, NR4A1, BCL2L1, BCL2L13
	0043066	Negative regulation of apoptosis	6	0.081	SKP2, TP53, HSPB1, BCL2L1, HSPA5, TAX1BP1
**Ubiquitination**	0006508	Proteolysis	15	0.007	CDH1, DERL2, SKP2, CDC20, PSMA2, PIAS4, HUWE1, BTBD1, WWP2, UBA1, UBE2K, CASP8, PIAS1, FBXO22, SPOP
	0006511	Ubiquitin-dependent protein catabolic process	7	0.004	PSMA2, CDH1, DERL2, UBE2K, SKP2, CDC20, FBXO22
	0004842	Ubiquitin-protein ligase activity	4	0.055	HUWE1, WWP2, UBE2K, FBXO22
**Intracellular transport**	0046907	Intracellular transport	11	0.010	CLTA, DERL2, NRBP1, SCYL1, MAP1S, LYST, TP53, BCL2L1, GGA1, RAB3IP, TOB1
	0045184	Establishment of protein localization	11	0.027	CLTA, DERL2, LYST, VPS52, TP53, PDIA4, GGA1, RAB3IP, VPS39, TOB1, KIF20A
	0016192	Vesicle-mediated transport	7	0.172	CLTA, NRBP1, SCYL1, LYST, GGA1, RAB3IP, KIF20A
	0048193	Golgi vesicle transport	4	0.053	CLTA, NRBP1, SCYL1, RAB3IP

Summary of the DAVID analysis gathering the E2 targets into functional families based on their Gene Ontology classification. We report enrichment p-values as it was calculated by DAVID. The asterisk (*) symbolizes manual inclusion into the transcription family.

#### Regulation of apoptosis

Multiple interactions between the E2 proteins and death or survival signaling pathways could be detected, through the targeting of 12 cellular proteins (table 2 and table S7). This is a common trend of viral proteins, since manipulations of cell death or survival pathways are key processes during viral infections. Generally, apoptosis is prevented in early phases of viral cycle to allow viral replication, and subsequent apoptotic induction occurs along with the production of viral particles [34]. The E2 proteins target both positive and negative regulators of apoptosis, suggesting a complex regulation of cell death pathways. Three apoptotic regulators were bound by all the E2 proteins (TP53, CASP8, TAX1BP1). Interestingly, for two of them, TP53 and CASP8, the binding of E2 may not have similar functional consequences according to HPV genotype. Indeed, apoptotic induction resulting from E2 binding to p53 was shown to be specific to HR-HPV [8]. The binding of LR-HPV E2 proteins to p53 detected here may either counteract p53 apoptotic functions or affect other p53 activities. Similarly, E2 binding to caspase 8 triggers apoptosis only for HR-HPV E2 proteins [35], since it depends on the cytoplasmic accumulation of the E2 proteins, which is specific to HR-HPV [7].

#### RNA processing

This family comprised nine proteins, of which six are involved in mRNA processing through the spliceosome (table 2, table S8). The interaction with splicing factors of the SR protein family was anticipated for β-types E2 proteins, through their hinge regions which contain long stretches of SR repeats [36]. Our results provide evidence that the targeting of SR-rich factors is conserved among all HPV genotypes. The α-type E2 proteins show greatly reduced levels of interaction, in accordance with the presence in their hinge of only short R-alternating sequences (Figure S4). Our results nevertheless suggest a conserved role of E2 in the regulation of RNA splicing. Of note, several E2 proteins were found to interact with PCBP1, a protein involved in the inhibition of translation of the late mRNA encoding the L2 capsid protein [37].

#### Ubiquitination

E2 targets were enriched in proteins involved in ubiquitination (table 2 and table S9). Some targets are general factors of the Ubiquitin Proteasome System, as the activating enzyme UBA1 or PSMA2 and POMP involved in formation of the 26S proteasome, possibly affecting the global process of proteasome-mediated protein degradation. Most E2 targets are, however, involved in the transfer of ubiquitin on substrates. They include ubiquitin ligases of the HECT domain family (HUWE1, WWP2), as well as substrate adaptors of Cullin-based ubiquitin ligase complexes (BTBD1, SPOP, CDC20, CDH1, FBX022). Given that E2 are ubiquitinated and degraded by the proteasome [38]–[40], some of these interactions probably mediate E2 degradation. Indeed, all E2 proteins were found to bind an adaptor of CUL3-based ubiquitin complexes (SPOP or BTBD1), in line with the involvement of these ligases in the degradation of HPV16 E2 protein [39]. Alternatively, E2 binding to Ub-ligases could have a functional impact by altering the degradation of their natural targets. This was shown for the binding of mucosal HR-HPVE2 to CDC20 and CDH1 (FZR1), which leads to the stabilization of cyclin B by inhibiting the “Anaphase Promoting Complex” ubiquitin ligase [9].

#### Intracellular trafficking

Unexpectedly, intracellular transport emerged from the HT-GPCA dataset as a functional family targeted by E2 (table 2 and table S10). A high proportion of E2 targets are involved in vesicle-mediated transport, affecting dynamics and maintenance of intracellular membranous organelles (table 2). Only one protein, VPS52, involved in traffic between the Golgi apparatus and endosomes, was bound by all the E2 proteins. Conversely, most of the E2 proteins interacted with several factors of this family. It highlights both a conserved and extensive targeting of intracellular trafficking factors, probably underlying novel E2 activities. This targeting is more concentrated on the Golgi apparatus, with 7 factors affecting this organelle (CLTA, SCYL1, VPS52, GGA1, KIF20A, NRBP1, RAB3IP). The Golgi is central in the translocation of processed viral antigens in association with type II MHC molecules. It might suggest that through this targeting, the E2 proteins would alter antigen presentation by infected keratinocytes. Surprisingly, E2 binds to several proteins involved in HPV entry pathways such as clathrin (CLTA), Rab-family proteins (RAB3IP), molecular motors (KIF20A), endosomal/lysosomal trafficking factors (VPS39) [41]. From this overlap, it is tempting to speculate that E2 may have a role in the early steps of infection. Only sparse information is available regarding E2 involvement in virus infectivity. In the BPV1 pseudovirion system, a study reported that E2 enhanced encapsidation of full-length viral DNA and may be packaged within the pseudovirion [42]. This was not corroborated by another study where E2 expression was not found to alter BPV1 pseudovirions production and infectivity [43]. The HPV pseudovirion system clearly works without E2 while requiring L2 [43]. One hypothesis would be that, in the context of a natural infection, the E2 protein is lying in the virion and could affect the nuclear translocation of viral genome in collaboration with L2. In conclusion, the functional targeting of intracellular trafficking possibly uncovers a novel biological function of E2, whose functional relevance requires further investigation.

### Functional and Biological Validation of a Subset of E2 Targets

A subset of E2 cellular targets was selected in order to provide further biological insight to some of the E2-host PPI identified from the HT-GPCA dataset.

#### HR-specific E2-cellular targets

We selected interactions that distinguished either HPV16 E2 or HPV18 E2 from other mucosal E2 proteins, since they might increase the understanding of the pathogenicity of these two viruses.

**Figure 6:**
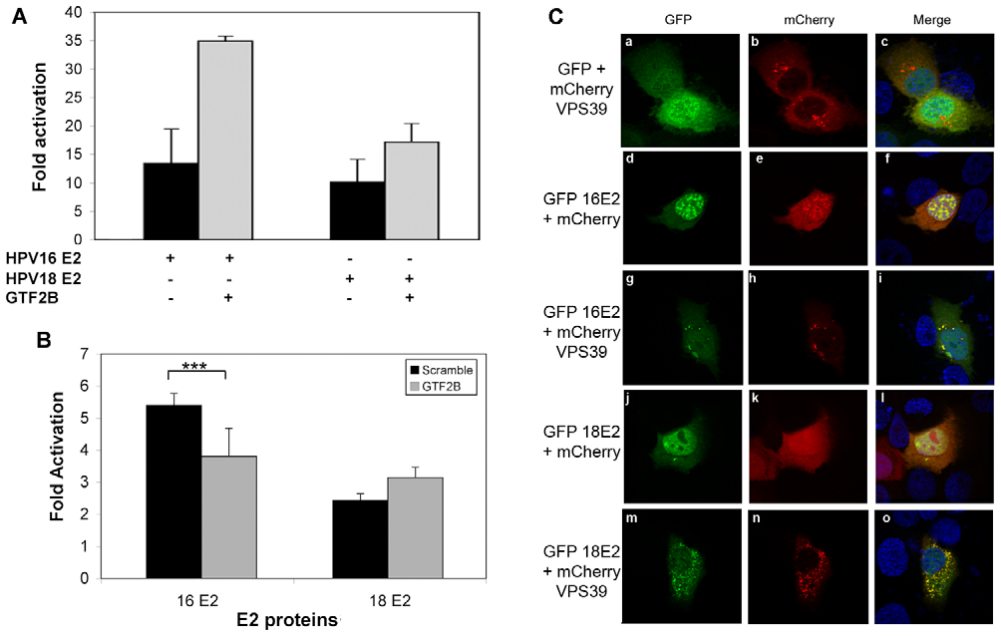
Validation of HR-specific interactions. (A) HeLa cells were transfected by pTK6E2BS-Luc reporter and HPV16 or HPV18 E2 expression plasmids. Where indicated, GTF2B was added. Fold activation is given relative to TK6E2BS-Luc in the absence of E2. (B) HeLa cells were transfected with a pool of four siRNA targeting GTF2B or control siRNA (Scramble). 48 h post silencing, pTK6E2BS reporter plasmid was transfected along with E2 expression plasmids. Results are given as a fold activation relative to TK6E2BS basal activity in the presence of the same siRNA. Experiments were performed in triplicate with each bar representing the mean ± SD. The stars (***) indicate a statistical significant difference between fold activation by 16E2 with a scramble siRNA or a GTF2B-directed siRNA directed (p-value<0,001) (C) HaCaT cells were co-transfected by GFP-E2 proteins from HPV16 or HPV18 and mCherry-VPS39. 24 h later, cells were fixed in 4% paraformaldehyde, stained with DAPI and subjected to fluorescence microscopy.

We first analyzed the impact of GTF2B on transcriptional activity of 16E2 in comparison to 18E2. Indeed, GTF2B binding is part of the PPI discriminating 16E2 from all the other tested mucosal HPV, including 18E2. Coexpression of GTF2B increased 2.6-fold the transcriptional activation of E2-responsive promoter by 16E2, while the effect on 18E2-mediated transactivation was minor (1.7 fold, Figure 6A). Accordingly, siRNA-mediated silencing of GTF2B impaired the activation of transcription by 16E2 but not by 18E2 (Figure 6B). These results substantiate both the functional relevance and the specificity of 16E2/GTF2B interaction.

Aiming to study a HR-specific PPI discriminating the 18E2 protein from all other mucosal E2, we chose VPS39, which plays a role in clustering and fusion of late endosomes and lysosomes. Both proteins were coexpressed in HaCaT keratinocytes fused to fluorescent tags, GFP (E2) and monomeric cherry (VPS39). As shown in figure 6C, VPS39 when expressed alone, exhibited a cytoplasmic distribution pattern in vesicles, in line with its association with lysosomes [44]. Coexpression with GFP-18E2 increased mcherry-VPS39 vesicles density, reminiscent of lysosomal clustering [44]. VPS39 vesicles were all labelled with GFP indicating a colocalization of 18E2 in these vesicles (Figure 6C). In contrast, 16E2 did not affect the density of VPS39 vesicle, despite some degree of colocalization. These results show that the specific interaction between 18E2 and VPS39 results in the clustering of VPS39 vesicles.

#### Targeting of intracellular trafficking factors

Given that E2 is primarily a nuclear transcription/replication factor, the targeting of cellular proteins involved in intracellular trafficking was a surprising aspect of our results. We therefore wished to visualize a subset of identified interactions using the colocalization assay previously described. Since it was the strongest interaction detected with 16E2 in this family, we first focused on the cellular protein VPS52 (Vacuolar Protein Sorting 52), a protein involved in vesicle trafficking from endosomes to the trans-Golgi network.

**Figure 7:**
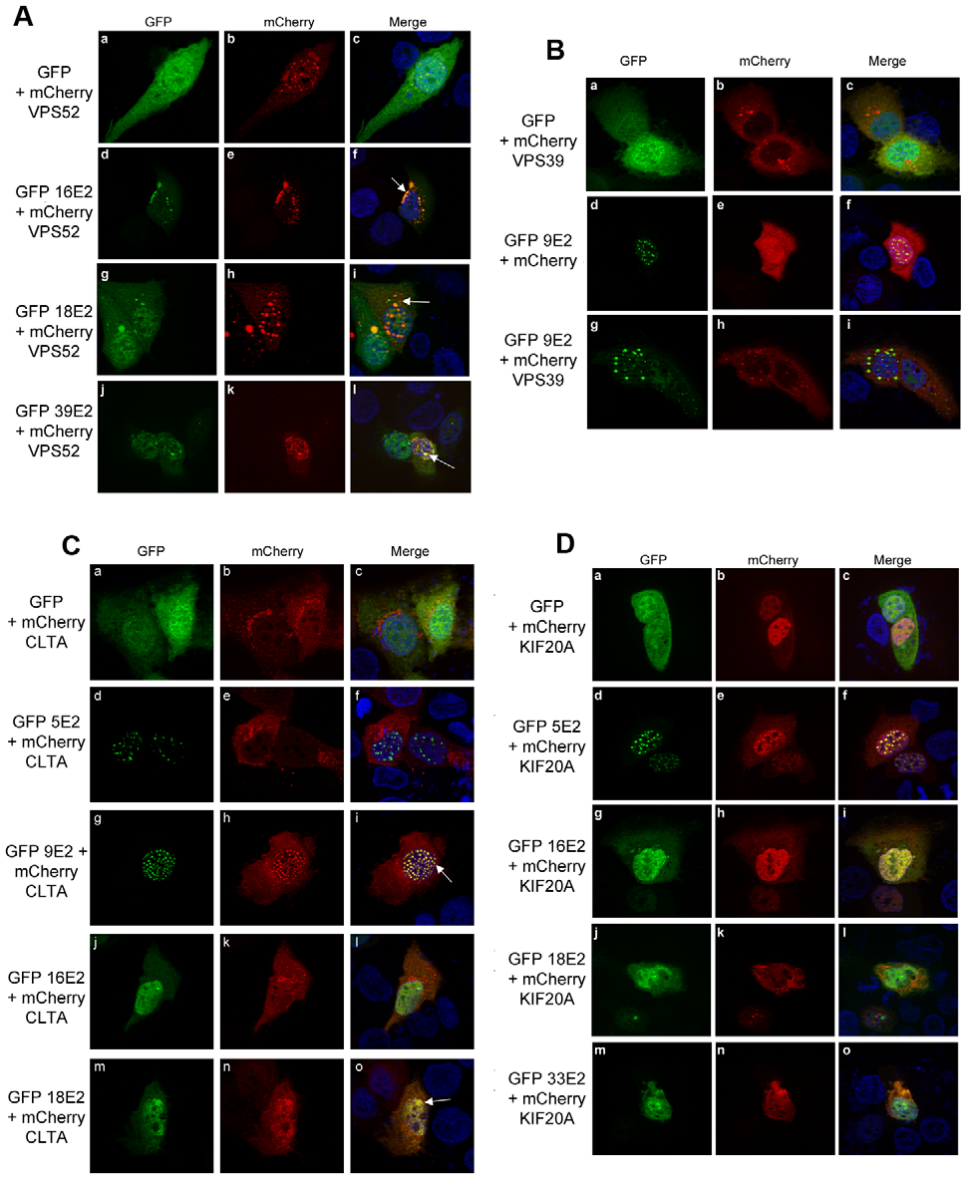
Fluorescence analysis of interactions between E2 proteins and intracellular transport proteins. (A–D) HaCaT cells were cotransfected with expression plasmids for the indicated GFP-E2 proteins and mCherry-VPS52 (A), mCherry-VPS39 (B), mCherry-CLTA (C), and mCherry-KIF20A(D). After fixation, the cells were subjected to fluorescence microscopy after counterstaining of the nucleus with DAPI.

Ectopically expressed VPS52 distributed in vesicles as described [45] (Figure 7A). When co-expressed with 16, 18 or 39 E2, a colocalization of VPS52 and E2 proteins could be observed (Figure 7A). In addition, VPS52 vesicles concentrated in a perinuclear region specifically in the presence of 16E2, which in turn massively redistributed in these vesicles. These observations are in good agreement with the HT-GPCA interaction data where 16E2/VPS52 NLR is the highest (table S10).

A similar redistribution was detected for 9E2 when co-expressed with VPS39 (figure 7B), in line with the VPS39 NLR profile in HT-GPCA. As shown in Figure 6C, VPS39 also interacts with 18E2. Interestingly, the impact of 18E2 and 9E2 expression on the pattern of VPS39 distribution varied, since vesicles clustering could only be observed in the presence of 18E2 (compare Figures 6C and 7B). The functional consequences of shared interactions may thus vary according to HPV genotypes, especially for the cutaneous and mucosal HPV which rely on more divergent pathogenesis.

Likewise, the interaction of clathrin light chain (CLTA) with 9 and 18E2 proteins, evidenced by colocalization, led to an increased nuclear accumulation of CLTA and induced different nuclear patterns (Figure 7C). No colocalization could be observed with 5 or 16 E2, in line with the HT-GPCA profiles.

We lastly studied KIF20A, a protein involved in the transport of Golgi membranes and associated vesicles along microtubules, and shown to localize both in the nucleus and in the cytoplasm where it can adopt a filamentous distribution [46]. Co-expression of KIF20A with a panel of E2 proteins confirmed the HT-GPCA results since all the E2 proteins interacting with KIF20A showed colocalization patterns (Figure 7D). When co-expressed with HPV5 E2, KIF20A strictly accumulated in 5E2-containing nuclear dots, while for other E2 proteins, in particular 18E2 and 33E2, colocalization could be observed both in the nucleus and cytoplasmic filaments.

Overall, the colocalization studies substantiate the targeting by E2 of proteins involved in cellular trafficking processes. In addition, they uncover differential effects of E2 binding on the distribution of targeted factors. This suggests that the E2 proteins may have various impacts on the intracellular trafficking, whose biological significance will clearly require further investigation.

Our comparative interactomics approach provides an unbiased mapping of E2-host protein-protein interactions and offers a unique opportunity to assess E2-host PPI profiles in relation to HPV tropism and pathogenic potential. The correlation between E2 interaction dendrograms and HPV phylogeny clearly demonstrates the reliability of the screening strategy, and suggests that E2 engages differential patterns of interaction. Accordingly, some interactions are discriminating the E2 proteins of HR-HPV from LR-HPV in the genital context. The targeting of cellular hubs accounts for a broad impact of E2 on the host cells. Analysis of E2-host PPI networks provides an overview of E2 biological functions across multiple HPV genotypes. It corroborates the essential role of E2 in the control of gene expression through regulation of transcription, which emerges as the prime target of the E2 proteins, and also through the regulation of RNA processing. In addition, the E2 proteins turn out to affect cell physiology through the targeting of apoptosis, ubiquination and intracellular trafficking. A striking feature of our results is the targeting of both positive and negative regulators of the same cell processes, suggesting dual roles of the E2 proteins. Further biological validations of a subset of identified PPI support interaction data, and provide evidence of a diversified functional impact of the E2 proteins on cellular processes. Overall, this study constitutes a framework for future functional investigations on E2 proteins and provides a solid basis to understand the role of E2 in HPV pathogenesis.

## Materials and Methods

### Plasmids

The 12 ORF encoding for the E2 proteins were amplified from viral genomic DNA corresponding to the different HPV genotypes, cloned by the gateway recombinational cloning system (Invitrogen) into the entry vector pDON207 (Invitrogen), and were listed in the ViralORFeome database [47]. The E2 ORFs were then transferred into gateway-compatible destination vectors pGBKT7-gw to generate E2-GAL4 DNA-binding domain fusion proteins for Y2H; pCI-Neo-FLuc-gw to generate Firefly luciferase-E2 fusions proteins for steady state levels measurement; pSPICA-N2-gw to generate proteins with amino acids 110 to 185 of the humanized *Gaussia princeps* luciferase in fusion with the N-terminus of E2 (GL2-E2 fusion proteins) for the High-Throughput *Gaussia princeps* Luciferase-based Complementation Assay (see [19] for construct details). Entry gateway plasmids for cellular partners were obtained either by PCR amplification from clones recovered by Y2H or from the human ORFeome resource (hORFeome v3.1). The cellular ORF were transferred into gateway-compatible destination vectors pSPICA-N1-gw to generate proteins fused at the N-terminus with the amino acids 18 to 109 of humanized *Gaussia* luciferase (GL1-fusion proteins). Mutagenesis of E2 proteins from HPV 16 and 18 was performed by PCR-directed mutagenesis method. The luciferase reporter (pTK6E2BS) driven by E2-responsive promoter contained 6 E2 binding sites upstream the minimal TK promoter. E2 BS sequences were as follows: (aACCGTTTTCGGTtaaACCGTTTTCGGTt )X3, designed after the study of Sanchez et al [21] to be optimal for the binding of a large panel of E2 proteins. The polymerase III-directed Renilla Luciferase plasmid (polIII-Ren) used as an internal control of transfection contained a 100-mer nucleotide encompassing the human Histone H1 promoter upstream of the Renilla ORF (hRluc).

### Yeast Two Hybrid

For yeast two hybrid screening, GAL4 DNA-binding domain-E2 fusion proteins, expressed from the pGBKT7 vector, were used to probe a human HaCaT cDNA library (Clontech), cloned in fusion with the GAL4 transcription activation domain in pACT2. Each independent screening was performed by mating pGBKT7-E2 transformed yeast strain AH109 (*MATa, trp1-901, leu2-3, 112, ura3-52, his3-200, gal4Δ, gal80Δ, LYS2 : : GAL1UASGAL1TATA-HIS3, GAL2UAS-GAL2TATA-ADE2, URA3 : : MEL1UAS-MEL1TATA-lacZ*) with Y187 strain (*MATα, ura3-52, his3-200, ade2-101, trp1-901, leu2-3, 112, gal4Δ, met–, gal80Δ, URA3 : : GAL1UAS-GAL1TATA-lacZ*) transformed with the HaCaT cDNA library. Mating was performed 4 hr at 30°C on plates of non-selective rich YCM media. The number of diploid cells generated was systematically evaluated to be at least 10 times higher the HaCaT cDNA library complexity (2.5×10^6^, Clontech).

Mated yeasts were grown on selective medium lacking tryptophan, leucine and histidine (SD-W-L-H), and supplemented with 3-aminotriazol according to the basal autoactivation test previously performed (see below). HaCaT cDNA sequences from positive colonies were PCR amplified and sequenced. Independent Y2H screens were repeated in the same way for each of the E2 protein until around 100 PPI could have been sequenced.

### Yeast Two-Hybrid Bait Basal Transactivation Test

Because bait constructs sometimes self-transactivate reporter genes, SD-W-L-H culture medium was supplemented with 3-aminotriazole (3-AT) in the Y2H screenings. Appropriate concentrations of this inhibitor were determined by growing bait strains (AH109 yeast strain transformed with each E2 bait) on SD-W-H culture medium supplemented with increasing concentrations of 3-AT. Concentrations of 3-AT ranging from 5 mM (for 33, 39, 18, 11, 5 and 8 E2) to 10 mM (for 1, 3, 6, 9, 32 and 16E2) were sufficient to counter the weak transactivation observed. This falls into the range of Clonetech standards.

### Analysis of Sequenced Y2H PPI (Interactor Sequence Tag or IST)

A bioinformatic pipeline was developed to assign each IST to its native human genome transcript. First, ISTs were filtered by using PHRED at a high quality score, sequence was extracted based on a sliding window of 30 bases which is successively shifted 10 bases until the average quality value from the window falls. A 30 bases motif from pACT2 linker was searched, sequences downstream of this motif were translated into peptides and aligned using BLASTP against human protein sequence databases from Ensembl (release 58 based on NCBI assembly 37), Uniprot and primate EMBL. Low-confidence alignments (E value > 10^−10^, identity < 80% and peptide length < 20 amino acids), frameshifted and premature STOP codon containing sequences were eliminated.

### High-Throughput *Gaussia princeps* Luciferase-Based Complementation Assay (HT-GPCA)

HEK-293T cells were seeded at 35,000 cells per well in 96-well plates. After 24 h, cells were transfected by linear PEI (polyethylenimine) with pSPICA-N2-E2 and pSPICA-N1-cellular protein constructs (100 ng each), for expression of the GL2-E2 and GL1-fusion proteins, where GL1 and GL2 are two inactive fragments of the *Gaussia princeps* luciferase. 10 ng of a CMV-firefly luciferase reporter plasmid was added to normalize for transfection efficiency. Cells were lysed 24 h post-transfection in 40 µL of Renilla luciferase lysis buffer (Promega) for 30 minutes. The *Gaussia princeps* luciferase activity was measured on 30 µL of total cell lysate by a luminometer Berthold Centro XS LB960 after injection of 100 µL of the Renilla luciferase substrate (Promega). Firefly luciferase was measured on the remaining 10 µl lysate with Firefly luciferase substrate. Gaussia Luciferase activity was reported to Firefly luciferase activity for each sample, giving a normalized Gaussia luminescence. Each normalized Gaussia luciferase activity was calculated from the mean of triplicate samples. For a given pair of proteins (A and B), the normalized Gaussia luminescence of cells coexpressing GL1-A+GL2-B proteins was divided by the sum of normalized Gaussia luminescence of each partner coexpressed with matched empty plasmid: GL1-A+GL2-B/(GL1-A +GL2) + (GL1 + GL2-B). This gave a Normalized Luminescence Ratio (NLR) corresponding to the reconstituted Gaussia luciferase activity, thus reflecting the level of interaction between protein pairs. See [19] for further details on the method.

### Analyses

Literature curated interaction (LCI) involving the E2 proteins were extracted from the VirHostNet [13], virusMINT [14] and PubMed databases. Interaction data analyses were performed using the R statistics package. Raw NLR interaction data were separated into categories in order to minimize the dispersion of NLR values. Cut-off thresholds of each category were determined with the goal of maintaining the same frequency distribution across all categories. An Euclidian distance matrix was calculated from the data categories using the “dist” function from R. The interaction dendrogram was calculated using the “complete” (UPGMA) linkage method from the “hclust” function from R. E2 protein sequences were clustered using the “phylip” package [48]. Protein distances were calculated with the “prodist” program, using default parameters. The phylogenetic dendrogram was generated with the “neighbor” program using the UPGMA method and default parameters. Both interaction and phylogenetic dendrograms were generated using JavaTreeView [49]. A Pearson correlation coefficient was calculated with the “cor” function in R using the cophenetic distances between both interaction and phylogenetic dendrogram to determine the closeness of the two dendrograms, The label order for the intensity data was then randomly changed to generate 100,000 random dendrograms. The cophenetic distance matrix for these randomized dendrograms was compared to the cophenetic distance matrix from the phylogenetic dendrogram with a Pearson correlation (“cor”) function from R. The p-value was calculated based on the number of standard deviations the correlation between the interaction dendrogram and the phylogenetic dendrogram was from the mean of the distribution of the correlation between the random and the phylogenetic dendrogram. A Cumulative Density Function of the randomized dataset was compared to a normal distribution generated by the R function ‘rnorm’ using the same mean and standard deviation from the randomized dataset to check the normality of the data.

The E2 interaction networks were generated with the cytoscape software [50] with interactions scoring positive in HT-GPCA (NLR above 3.5). The degree of each cellular protein in both E2 and HPRD-based human interactomes was extracted from cytoscape. To determine the overrepresented GO (Gene Ontology) terms in the interaction dataset and to evaluate the gathering of E2 targets by functional categories, we used the DAVID bioinformatic database [32]. P-values were generated by DAVID.

### Transactivation Assay

293T cells were plated at 35,000 cells per well in 96-well plates and transfected 24 h later by linear PEI with 25 ng of pTK6E2BS E2 responsive reporter plasmid, 10 ng of the polIII-Ren as internal control for transfection efficiency, and 100 ng of GL2-E2 fusion proteins or empty GL2 plasmid. To assess the effect of GTF2B, HeLa cells plated in 12-well plates were transfected by linear PEI with 100 ng of pTK6E2BS, 10 ng polIII-Ren, 100 ng of mCherry-fused E2 or mCherry expressing plasmids, and either 1 µg of GTF2B expressed from pCI Neo or of empty pCI Neo (Promega). 30 h post transfection, cells were lysed in Passive lysis buffer according to manufacturer's instructions and luciferase activity was measured with Dual Glo Buffer (Promega). Results are given as the mean of three independent tests ± SD (errors bars).

### Fluorescence Assay

HaCaT cells grown in coverslip were co-transfected by linear PEI with expression plasmids for GFP-fused E2 proteins (3 µg) and Cherry-fused cellular proteins (1 µg). 24 h post transfection, cells were fixed with 4% paraformaldehyde for 30 min, washed in PBS, and incubated with DAPI for 30 min. Cells were mounted with CitiFluor. Fluorescent Images were acquired using a ZEISS Apotome microscope.

### siRNA Assay

7,500 HeLa cells were reverse transfected by INTERFERin (Polyplus-Transfection) with 1.75 picomole of a pool of four siRNA targeting GTF2B (from Qiagen bank Human Whole Genome siRNA Set V4.1), and plated in 96-well plates. 2 scrambled siRNA (ref 1027310, Qiagen) were used as negative controls. 48 h later, 20 ng of Cherry-E2 expression plasmids were transfected by linear PEI along with the 25 ng of pTK6E2BS reporter and 10 ng of polIII-Ren as internal control for transfection efficacy and cell viability. 24 h post transfection, cells were lysed in passive lysis buffer according to manufacturer's instructions (Promega). Firefly and Renilla luciferase were measured on a Berthold Centro luminometer to generate a Luciferase/Renilla ratio, each transfection was tested in triplicates with each bar representing the mean ± SD. Results are given as fold activation of TKE2BS by E2 in the presence of the siRNA, calculated relative to TKE2BS activity without E2. P-values were calculated by a Student statistical test.

## Supporting Information

Figure S1 **Schematic comparison of Y2H and HT-GPCA datasets.** Summary of the interactions detection, selection and validation by Y2H and HT-GPCA.
(TIF)

Figure S2 **Comparison of different parameters for dendrograms generation.** Different parameters of distance and linkage were tested to generate the interaction-based dendrograms and are indicated on the left. The corresponding tree structure is represented and compared to phylogenetic tree generated with the E2 protein sequences. The cophenetic correlation coefficient is specified for each combination of dendrogram.
(TIF)

Figure S3 **Criteria for selection of functional families.** A DAVID analysis was performed on the targets of the E2 proteins. Several parameters have been taken into account for the selection of the five most pertinent functional families: low p-value (A), high enrichment score (B) and high prevalence (C).
(TIF)

Figure S4 **Alignment of the E2 hinge region amino acids sequences of 12 HPV.** The arginine (R) residues and the serine residues (S) are highlighted in red and green respectively. The HPV genotype is indicated on the left of each row.
(TIF)

Table S1 **Literature curated interactions (LCI).** List of interactions found for the HPV E2 proteins in the VirHostNet and virusMINT and PubMed databases. The circles (°) represent interactions only found by literature mining. The number 1 symbolizes a demonstrated interaction, while 0 stands for a non-detected interaction. The number of LCE2-PPI represented in our GS dataset is indicated.
(XLS)

Table S2 **Comparison of Y2H with published E2-PPI.** Cellular proteins from the Y2H dataset that were previously identified as E2 interacting partners. Numbers represent Y2H hits and asterisks (*) represent previously identified interactions (LCE2-PPI).
(XLS)

Table S3 **Y2H data.** List of selected Y2H sequenced PPI (or Interactor Sequence tag, IST) detected for each of the E2 protein.
(XLS)

Table S4 **HT-GPCA interaction dataset between E2 proteins and the gold standards.** Matrix of Normalized Luminescence Ratio (NLR) between the 24 gold standards (positive controls) and the 12 E2 proteins. In bold are represented the gold standards identified in the Y2H screen. The asterisks (*) represent interactions described in the literature (LCE2-PPI).
(XLS)

Table S5 **Total HT-GPCA interaction dataset.** Table representing the Normalized Luminescence Ratio (NLR) calculated for the 1,452 interactions (12 E2 proteins and 121 cellular proteins) tested. In bold, the gold standards.
(XLS)

Table S6 **Transcription regulation family.** Interaction scoring obtained by HT-GPCA.
(XLS)

Table S7 **Apoptosis family.** Interaction scoring obtained by HT-GPCA.
(XLS)

Table S8 **RNA processing family.** Interaction scoring obtained by HT-GPCA.
(XLS)

Table S9 **Ubiquitination family.** Interaction scoring obtained by HT-GPCA.
(XLS)

Table S10 **Intracellular transport family.** Interaction scoring obtained by HT-GPCA.
(XLS)
