# Supplementary figures and images for: Large Scale Genotype Comparison of Human Papillomavirus E2-Host Interaction Networks Provides New Insights for E2 Molecular Functions
Source: PLoS Pathog. 2012 Jun 28;8(6):e1002761. doi: 10.1371/journal.ppat.1002761 (PMC3386243; doi:10.1371/journal.ppat.1002761)

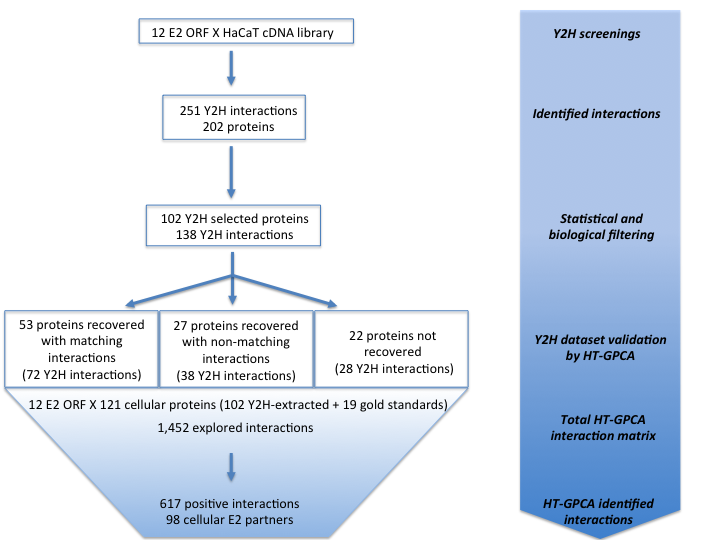

Supplement: Figure S1 — Schematic comparison of Y2H and HT-GPCA datasets. Summary of the interactions detection, selection and validation by Y2H and HT-GPCA. (TIF) [file ppat.1002761.s001.tif]

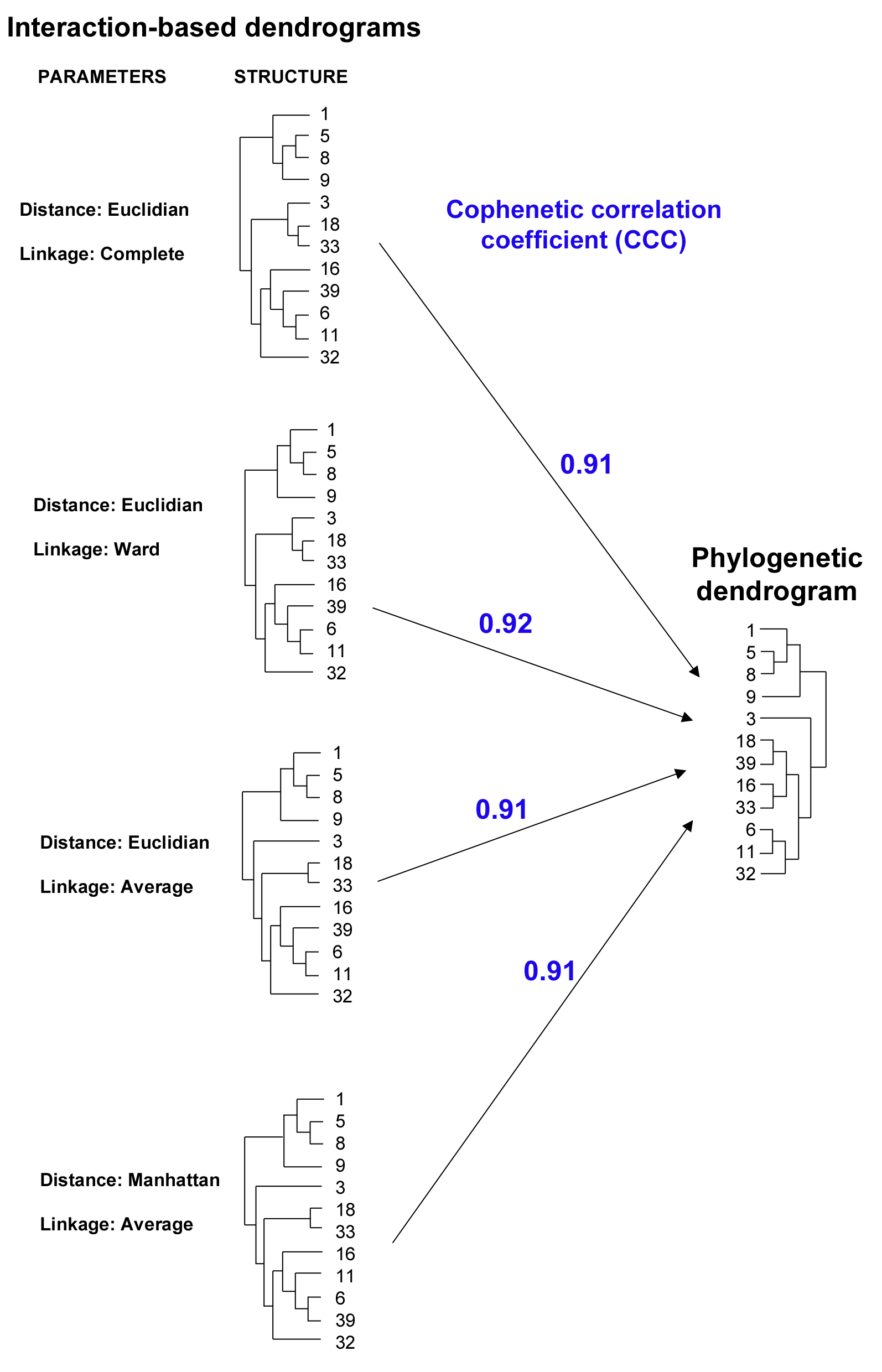

Supplement: Figure S2 — Comparison of different parameters for dendrograms generation. Different parameters of distance and linkage were tested to generate the interaction-based dendrograms and are indicated on the left. The corresponding tree structure is represented and compared to phylogenetic tree generated with the E2 protein sequences. The cophenetic correlation coefficient is specified for each combination of dendrogram. (TIF) [file ppat.1002761.s002.tif]

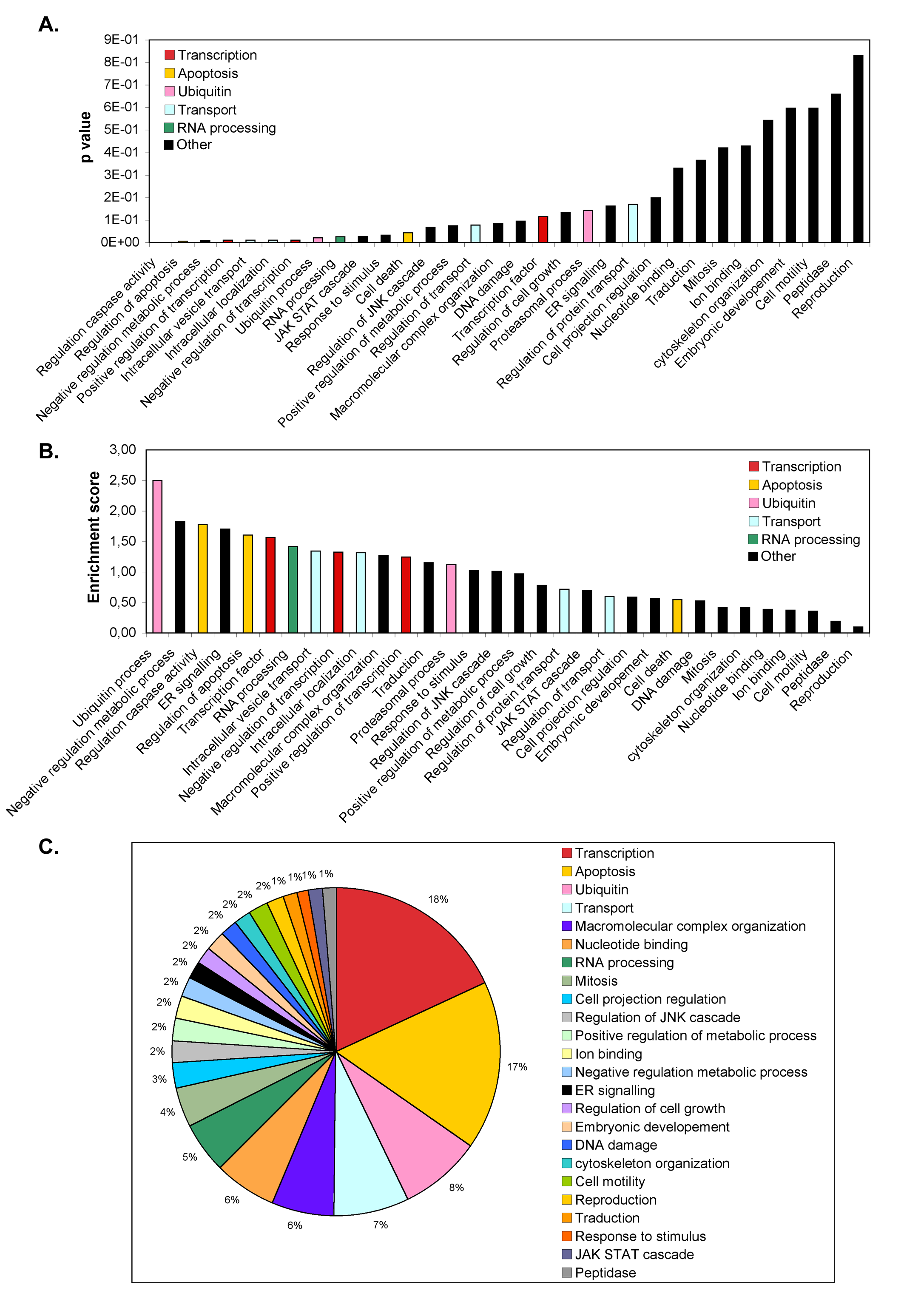

Supplement: Figure S3 — Criteria for selection of functional families. A DAVID analysis was performed on the targets of the E2 proteins. Several parameters have been taken into account for the selection of the five most pertinent functional families: low p-value (A), high enrichment score (B) and high prevalence (C). (TIF) [file ppat.1002761.s003.tif]

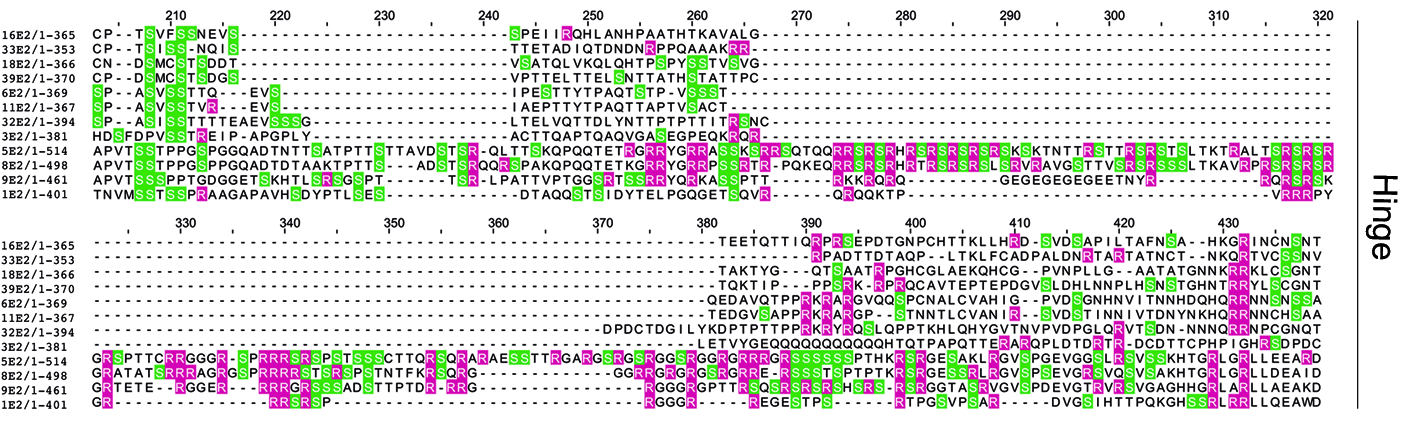

Supplement: Figure S4 — Alignment of the E2 hinge region amino acids sequences of 12 HPV. The arginine (R) residues and the serine residues (S) are highlighted in red and green respectively. The HPV genotype is indicated on the left of each row. (TIF) [file ppat.1002761.s004.tif]
